# Preparation of Messenger RNA Nanomicelles via Non-Cytotoxic PEG-Polyamine Nanocomplex for Intracerebroventicular Delivery: A Proof-of-Concept Study in Mouse Models

**DOI:** 10.3390/nano9010067

**Published:** 2019-01-05

**Authors:** Long Yi Chan, Yit Lung Khung, Chin-Yu Lin

**Affiliations:** 1 Institute of New Drug Development, China Medical University, No.91 Hsueh-Shih Road, Taichung 40402, Taiwan; lyc0429@mail.cmu.edu.tw; 2 Department of Biological Science and Technology, China Medical University, No.91 Hsueh-Shih Road, Taichung 40402, Taiwan; 3 Tsuzuki Institute for Traditional Medicine, Collage of Pharmacy, China Medical University, No.91 Hsueh-Shih Road, Taichung 40402, Taiwan

**Keywords:** mRNA, polyplex nanomicelle, intracerebroventricular injection, aminoethylene side chains

## Abstract

The specific delivery of messenger RNA (mRNA) is an excellent alternative to plasmid DNA, due to the latter’s potential risk for random integration into the host genome. In this study, we propose the use of specially tailored polyplex nanomicelles for the intravenous delivery of mRNA into the brain of mice. In brief, along the backbone of a polyaspartamide polymer that is terminated with a 42k Polyethylene glycol chain (PEG), aminoethylene-repeating groups (two, three, and four units, respectively) were conjugated to side-chains to promote electrostatic interactions with mRNA. This structural configuration would ultimately condense into a polyplex nanomicelle ranging between 24 and 34 nm, as was confirmed by transmission electron microscopy (TEM) and dynamic light scattering (DLS) while the chemistry of the synthesis was validated through NMR analysis. Subsequently, we hypothesized an important correlation pertaining to the role of hydrogen bonding between the interaction of polyamine and mRNA in due course. As a proof of concept, we encapsulated the *luciferase* (*Luc2*) mRNA as a reporter gene through in vitro transcription (IVT) and subsequently infused the polyplex nanomicelles into mouse brains via an intracerebroventricular (ICV) injection to bypass the blood–brain barriers (BBB). Data revealed that PEGylated polyplex nanomicelles possessing four repeating units of aminoethylene groups had exhibited the best *Luc2* mRNA delivery efficiency with no significant immune response registered.

## 1. Introduction

While the potency of mRNA-based therapeutics is highly undisputed, the mode of delivery is often the main hindering factor for its limited use [1,2]. *In-vitro* strategies, although useful in demonstrating the viability of mRNA, do not necessarily translate into the feasibility at clinical level, as cytotoxicity is often a major concern. This is especially obvious with cationic liposomal delivery platforms with highly documented cytotoxicity profiles [3,4]. Furthermore, it is often deemed necessary to provide shielding on the naked mRNA as they tend to be highly susceptible to biodegradative attacks in vivo and may at times trigger undesirable immunogenic responses from the host [5]. Hence, these issues have gradually given rise to a greater emphasis on designing non-cytotoxic and smarter delivery systems that are both biodegradable as well as biocompatible.

Of the many nanoparticulate platforms proposed so far in literature for nucleic acids and macromolecules [6,7,8], one interesting concept—widely advocated by Kataoka’s group in recent years —is on the use of Polyamine as a carrier for packaging and delivering mRNA [9,10]. In brief, by relying on the electrostatic interaction of positively charged amino (NH) moieties and the negatively charged ribonucleotides, mRNA can be condensed to form a polyplex configuration and be subsequently delivered into cellular targets. What sets this strategy apart from other conventional linear polyionic complexes is the presentation of the short cationic NH tethers that extend from the side-chains of the polymer rather than incorporating them along the linear backbone of the polymer, and this was found to have substantially lower cytotoxicity compared with other chemical arrangements [11,12]. This was frequently demonstrated on the framework of polyaspartamide-derivatized polymers and was found to be highly efficient for mRNA condensation as well as for delivery. In fact, the authors had also often addressed the concepts of the mRNA-polyplex escaping endosomal entrapment, which was essential to ensure a proper delivery into intracellular space. Under normal cell culture conditions with linear polycations, the degree of protonation on the NH was reported to have increased with the reduction of pH, and as the pH of endosomal entrapment was at 5, an endosomal disruption and a mRNA release into the intracellular space would have occurred via an increment of osmotic pressure within the endosomal space [13]. However, such polycationic polymers may often interfere with cellular plasma membranes and can result in membrane damage, leading to inherent cytotoxicity [14,15]. By localizing the domains of the protonation at the side chains of the polymer, Kataoka et al. argued that the polymeric mainframe would help to shield the protonic charge from interfering with the cellular membrane right from the onset, and that the eventual transition towards an envelopment within the endosomal space would build up sufficient osmotic pressure to liberate the contents within the intracellular. This can effectively help to address some issues regarding cytotoxicity, and such a strategy was found to have a profound effect in reducing collateral damage to tissues during the administration of mRNA.

One interesting improvement on such a mRNA delivery model would be the incorporation of PEG chains to the modified polyasparatimide framework, as PEG can confer ‘stealth-like’ properties to the overall polyplex nanocomposite [16,17,18]. This strategy is deemed logical, as such synergistic models can potentially help to address the issue of immunogenic response in vivo and may also improve on the overall stability of this delivery system. However, while there had been reports on using PEG conjugation to this system in vitro, there had been very little to show for in terms of clinical application to help promote the applicability of this polyplex model [19]. Hence, in this paper, a PEG chain was introduced to a short chain of a modified polyasparatimide based on previous models and this polyplex carrying *Luc2* mRNA was administered directly to brain region of mouse (animal model) to demonstrate the viability of such a modified polyplex system. In brief, via a copolymerization approach, a 42k PEG was covalently attached to asparatimide chains via aminolysis, and the sequential chemistry of the copolymer was carefull analyzed via NMR analysis. The visual examination of these nanocomplexes were in turn confirmed with TEM and DLS while the efficiency of the mRNA encapsulation was studied via confocal microscopy. There are significant advantages in such PEG-conjugating system unlike conventional polymerization processes: the chain length of both PEG and polyaspartamide can be highly controlled as well as fine-tuned and this allows for a precise ordering of size of the final nanocomplex. This report also represents one of the early attempts to administer such PEG-based nanocomplex/mRNA hybrid vesicles for the treatment of brain disorders in animal models.

## 2. Methods and Materials

### 2.1. Synthesis of Block Copolymer and NMR Analysis

**Figure 1:**
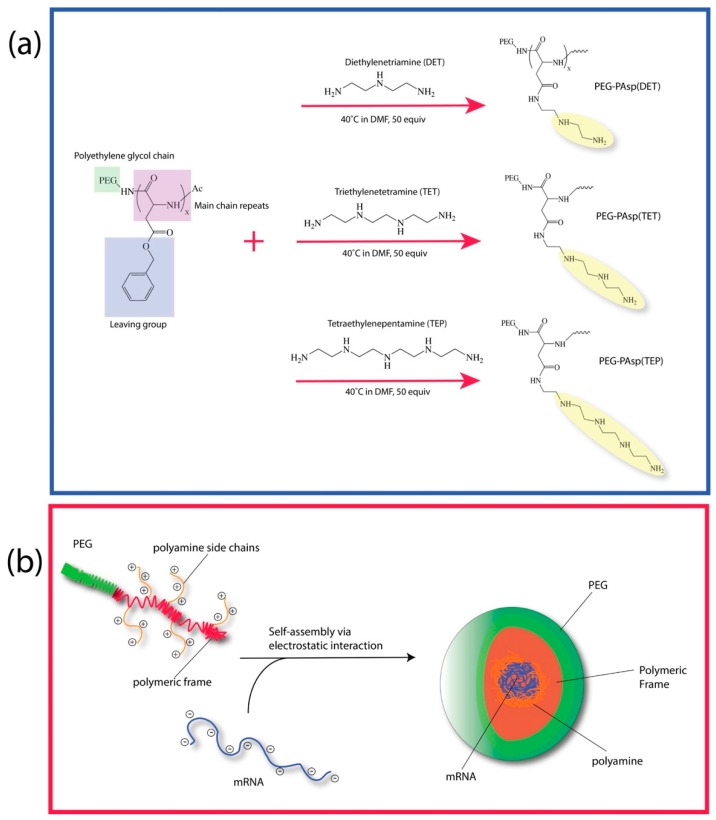
(**a**) Graphical illustration on the assembly of the polyamine of various length (DET, TET, and TEP) respective via the aminolysis of the benzylic alcohol side chains and (**b**) the subsequent condensation with mRNA into nanomicelles in physiological environment.

The PEGylated block catiomer was synthesized, as previously reported, with a slight modification [20]. In brief, the aminolysis of benzyl groups in the side-chain of a poly(benzyl l-aspartate) (PBLA) segment of a PEG-PBLA block copolymer was first performed to generate N-substituted polyaspartamides (Figure 1a) to carry 2, 3, and 4 repeating units of aminoethylene on the side chain. The aminolysis of the PEG-PBLA process to incorporate repeating aminoethylene units as a side-chain was performed in dry dimethylformamide (DMF) (Sigma-Aldrich, St. Louis, MO, USA) at 40 °C with either diethylenetriamine (DET), triethylenetetramine (TET), or tetraethylenepentamine (TEP) (Sigma-Aldrich, St. Louis, MO, USA) for 24 h in the presence of a molar excess (50 equiv relative to benzyl groups) of an aminoethylene donor. We then synthesized PEG (M.W. = 42,000)-poly{*N*-[*N′*-(2-aminoethyl)-2-aminoethyl]aspartamide}, possessing 2 repeating aminoethylene units, abbreviated as PEG-PAsp(DET); PEG (M.W. = 42,000)-poly(*N*-{*N′*-[*N″*-(2-aminoethyl)-2-aminoethyl]-2-aminoethyl}aspartamide), possessing 3 repeating aminoethylene units, abbreviated as PEG-PAsp(TET); and PEG (M.W. = 42,000)-poly[*N-*(*N′*-{*N″*-[*N‴*-(2-aminoethyl)-2-aminoethyl]-2-aminoethyl}2-aminoethyl)aspartamide], possessing 4 repeating aminoethylene units, abbreviated as PEG-PAsp (TEP) (Figure 1a). The degree of substitution of the benzyl group was determined by ^1^H NMR analysis (400 MHz, D_2_O) with DET to be approximately 56%, and the substitution with TET moiety was determined to be approximately 60%, and finally the substitution with a TEP moiety was determined to be approximately 62% by ^1^H NMR analysis (400 MHz, D_2_O).

### 2.2. Construction of Vector for In Vitro Transcription (IVT) and Preparation of IVT mRNA

The IVT vector used for the production of *Luc2* mRNA was sub-cloned from pDNAs encoding photinus pyralis luciferase (pGL4; Promega Corporation, Madison, WI, USA) and with the cDNA fragment inserted into the pSP73 vector (Promega Corporation) under the control of a T7 promoter containing 120 bps of chemically synthesized poly(d(A/T) fragments at the downstream of the cDNA region [21]. Then, the vectors were linearized with BsmBI (Figure S1A), blunted with T4 DNA polymerase, purified with gel electrophoresis, and then served as templates for IVT using the mMESSAGE mMACHINE T7 Ultra Kit (Thermo Fisher Scientific, USA) to generate mRNA. The mRNAs encoding green fluorescence protein (GFP) was similarly constructed from the vectors encoding green fluorescence protein (pEGFP-1; Clontech Corporation, Mountain View, CA, USA). Prior to the experiments, all transcribed mRNAs were purified with an RNeasy Mini kit (Qiagen, Venlo, The Netherlands) and analyzed for size and purity with the Agilent RNA 6000 Nano Assay on a BioAnalyzer 2100 (Agilent Technologies, Santa Clara, CA, USA) (Figure S1B).

### 2.3. Intracerebrovetricular (ICV) Administration

To prepare the polyplex nanomicelles, PEGylated catiomers and *Luc2* mRNA were separately dissolved in 10 mM of a hypotonic HEPES buffer that temporarily loosens up the epithelial junctions so as to facilitate for the absorption of drugs and genes [22,23]. The two portions were then combined in a volume ratio of 1:2 and adjusted the ratio of amino groups in catiomers over phosphate in mRNA (N/P ratio) to 3. All transgenes, either encapsulated within the polyplex nanomicelles or non-encapsulated were prepared and immediately applied on ICV infusion. Meanwhile, 8 to 12-weeks-old female SPF ICR mice were anesthetized by inhalation of 2.5% isoflurane (Abbott), fixed and placed in a prone position on the stereotaxic instrument (Narishige Group, Setagaya-ku, Tokyo, Japan). A 1.0- to 1.5-cm sagittal incision was made on the scalp of each mouse with the calvarium exposed by blunt dissection. A tiny parietal hole was created on the sagittal suture of skull (1.5 mm posterior to the bregma) perpendicularly by a 1-mm diameter trephine bur. A 3-μg transgene mixture in a total 6-μL volume was then delivered to mouse via the 3rd ventricle at a depth of 3.0 mm ventral to the surface of skull, according to the brain atlas as provided by Franklins and Paxinos [24], using a Hamilton syringe connected to a stereotaxic micromanipulator (Narishige Group, Japan) at a rate of ≈1.2 μL/min. Subsequently, the incision was closed using 4-0 nylon sutures. All animal experiments were approved by the China Medical University Committee for the Use and Care of Animals.

### 2.4. Luciferase Expression Measurement by Bioluminescence Assay

To measure firefly *Luc2* in vivo expression, a d-Luciferin substrate (Promega, USA) was dissolved in PBS and adjusted to a final concentration of 15 mg/mL. Bioluminescence was then measured with an IVIS imaging system (Xenogen, Alameda, CA, USA) 5 min after intraperitoneally injecting 200 μL of the d-Luciferin solution, and bioluminescence signals in the brain region were analyzed by background subtraction using Living Image Software (Xenogen, Alameda, CA, USA).

### 2.5. Histological Examination

To track the *GFP* mRNA delivery in mouse brain, 3 μg of mRNA were incorporated into PEG-PAsp(TEP) for an ICV infusion. After 24 h, the mice were sacrificed, perfused with PBS, and their brains were fixed with 4% of PFA in PBS, immersed with a gradient sucrose to 30% concentration and embedded in OTC for frozen sectioning. Sagittal serial sections (5 μm in thickness) from bregma to lateral ≈3 mm site were prepared (CM3050S, Leica, Wetzlar, Germany) for immunohistochemical (IHC) staining by standard protocol. In brief, slides were washed, blocked, and immune-stained with rabbit an anti-GFP 1st antibody (Invitrogen, Carlsbad, CA, USA) at 4 °C o/n and an Alexa 488-conjugated goat anti-rabbit 2nd antibody (Jackson ImmunoResearch Inc., West Grove, PA, USA) at RT 1 h, after which they were counter-stained with DAPI (VECTASHIELD^®^, VECTOR Laboratories, Inc., Burlingame, CA, USA) and observed under fluorescence microscopy (Carl Zeiss, Oberkochen Germany).

### 2.6. Quantitative REAL Time PCR (qRT-PCR) to Examine the Immune Response after Nanomicelle ICV Administration

To demonstrate the reduced systemic immune responses of mRNA in animal administration attributed from nanomicelle protection, we applied naked *Luc2* mRNA or nanomicelle-protected *Luc2* mRNA in ICV administration, followed by brain total RNA extraction, cDNA synthesis, and qRT-PCR. In brief, 3 μg of naked *Luc2* mRNA or PEG-PAsp(TEP) block-copolymer-protected *Luc2* mRNA were respectively ICV injected into the mouse brain. At 1, 4, 24, and 48 h post-injection time, mice were perfused with PBS, after which the whole brain was removed and immediately immersed in liquid nitrogen, then homogenized with 750 μL of buffer RLT (Qiagen) by a multi-bead shocker (Yasui Kikai Corporation, Osaka, Japan) at 2500 rpm for 30 s. A 100 μL, the brain lysate was subjected to total RNA isolation using an RNeasy Mini Kit (Qiagen, Geschäftsführer, Germany) in accordance with the manufacturer’s instruction. The cDNA was then converted by a iScript gDNA Clear cDNA Synthesis Kit (BioRad, Hercules, CA, USA). Gene expression of cytokines and interferons were analyzed by RT-PCR using an ABI QuntaStudio 3 Sequence Detector (Applied Biosystems, Foster City, CA, USA), and TaqMan Gene Expression Assays (Applied Biosystems, Mm00446190_m1 for interleukin (IL)-6, Mm00439552_s1 for interferon (INF)-β1, and Mm00607939 for β-actin).

### 2.7. Statistical Analysis

Results are expressed as the means ± standard error of mean (SEM). Statistical comparisons were performed by two-way analysis of variance (ANOVA), and *p* values < 0.05 were considered significant. All data are representative of at least three independent experiments.

## 3. Results and Discussion

### 3.1. Synthesis of Block Copolymer and Preparation of Polyplex Nanomicelles

As shown in Figure 1a, along the backbone frame of polyaspartic acid, three polyamine molecules of different lengths—Diethylenetriamine (DET), Triethylenetetramine (TET) and Tetraethylenepentamine (TEP)—were covalently grafted via a nucleophilic aminolysis of benzyl alcohol protective moiety. To further improve on the stability of the overall polymeric complex, polyethylene glycol (PEG) chain with a molecular weight of 42k Dalton was introduced to the C-terminus end of the polyaspartic frame. It had been previously reported in the literature that the presence of the polyamine would condense mRNA via electrostatic/hydrogen bonding interaction [9,20]. Under normal physiological environments, it is hypothesized that these polymeric complexes would organize to condense the mRNA load in the interior region while exposing the more hydrophilic PEG domains to the peripheral region. This condensation issue would ultimately restructure itself into the form of micellar nanoarchitecture, as shown in Figure 1b. Furthermore, the addition of PEG was also intended to help provide shielding to the interiorly trapped mRNA against any potential degradation in the physiological environment.

### 3.2. NMR Analysis of Block Copolymer

**Figure 2:**
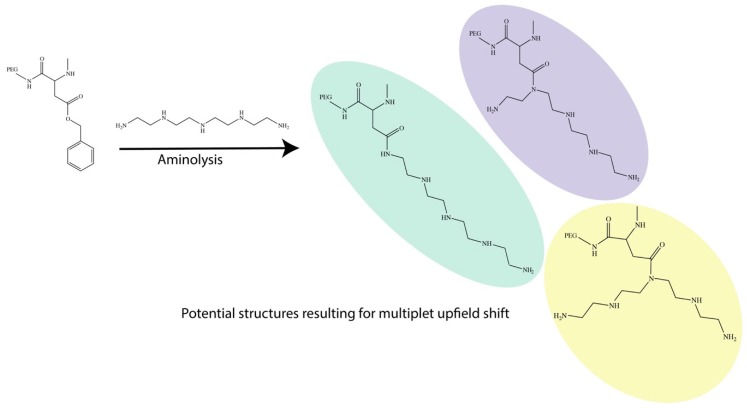
Due to the nature of the nucleophilic substitution of the benzylic alcohol, we postulated the range of different reaction sites on the polyamine as exemplified by the reaction of TEP, resulting in possible differences in extension of arms.

An ^1^H-NMR analysis is an important tool to evaluate the overall outcome of the aminolysis and is as shown in Supplementary Figures S2–S4 for all the polyamine complexes. Interestingly, for the DET polymeric complex, we were able to obtain a well-defined peak position, as shown in Figures S1–S3, while TET and TEP had various overlapping multiplet peaks from 2.5–5.0 ppm. It is also important to note that all NMR was acquired under Deuterium Oxide (D_2_O) in order to quench all signals from NH and OH so as to reduce the overall complexity during the analysis. Due to the polymeric nature, we noticed that there were many overlapping signatures, and hence specific assignment relative to the peak splitting was technically challenging. However, from the ^1^H-NMR analysis, we observed that the aminolysis reaction between the benzylic alcohol-protected polyaspartic acid and the polyamine can potentially yield various sites of conjugation, rather than the nominally intended NH_2_ tail ends (Figure 2). This was thought to have significant consequences on our subsequent observation of mRNA delivery efficiency, although such observations do corroborate with previous studies. Firstly, the events of the aminolysis is one of a nucleophilic nature with lone pair electrons from the amine, and it is hence not practical to expect that only the NH_2_ tail group can react to displace the benzylic alcohol. On the basis of this, we expected the reaction to have also occurred at any secondary amine position on the polyamine chain and on a single polyaspartic chain, we expected the reaction to be relatively random. This, in turn, gave rise to a polymer structure with a highly random insertion of polyamine, subsequently resulting in multiple overlapping upfield peaks; they were also found to be increasingly more chaotic and with an increased polyamine chain length [25]. Nonetheless, we found that these spectrums were in full agreement with numerous observations made by Kataoka’s group in the past [26,27].

### 3.3. Physical Property Analysis of Polyplex Nanomicelles

**Figure 3:**
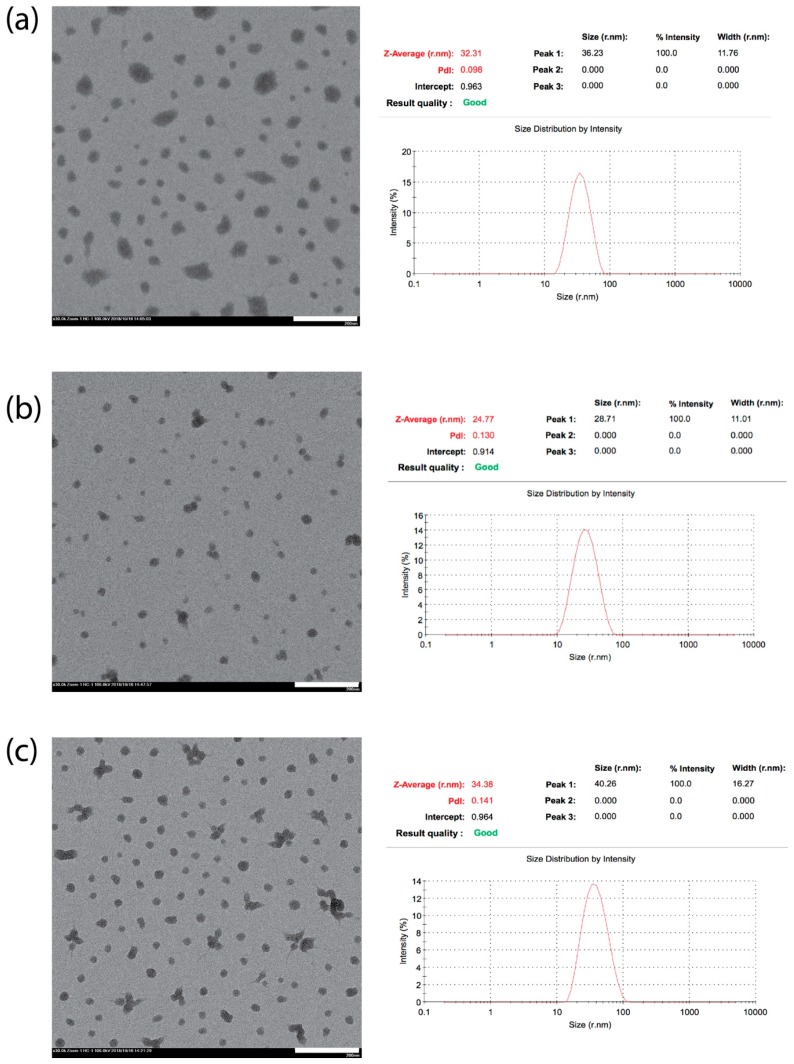
TEM and DLS analysis of *Lux-2* mRNA condensed nanomicelle of (**a**) PEG-PAsp(DET), (**b**) PEG-PAsp(TET) and (**c**) PEG-PAsp(TEP) with the white bar representing 200 nm. Both DET and TEP were found to be marginally larger (31–35 nm) compared to TET (~24 nm), and this has been attributed to the uneven extension of chain arm upon aminolysis.

In terms of utilizing this polyplex construct, it was more important to deduce the overall morphology upon encapsulation with mRNA, and as shown in Figure 3, we had performed both TEM and DLS to determine the precise sizing and shape of these particles. By correlating both sets of data, we observed that for DET and TEP, the sizing of the micelles were found to range between 31–35 nm in general, while TET had been observed to be generally smaller, at ~24 nm. Although at first glimpse, the similarity in chemistry should dictate that all polyplex nanomicelles would possess a relatively identical sizing range, it is more likely that TET conjugation of the polyamine may have contributed to the overall disparity in sizing.

**Figure 4:**
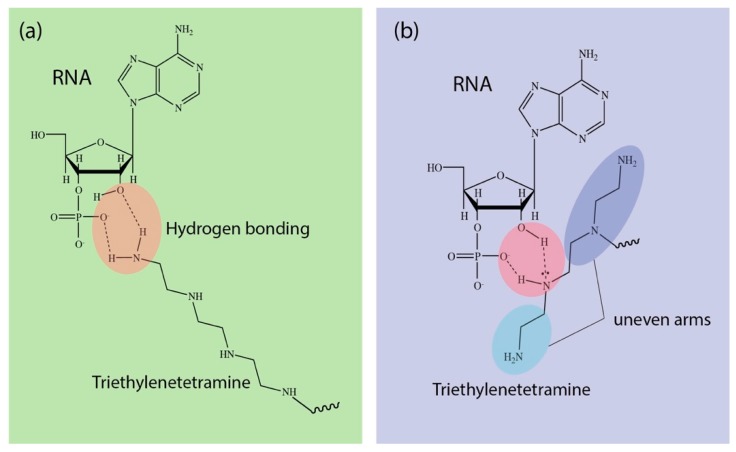
Hydrogen bonding of TET via (**a**) tail group amine and (**b**) secondary amine resulting in “uneven” chain arms.

As mentioned earlier from the NMR analysis, due to the randomness of the reaction, a four-amine group carrying TET could either react at the tail end of the chain or at any random secondary amine site. What is more important is that this random reaction would give rise to “unevenness” in the polyamine chain arm, especially for nucleophilic substitution at the secondary amine position. This may have a profound effect on the interaction with mRNA, as it has been well-established that polyamine may interact via both strong electrostatic as well as a hydrogen bonding interaction with the carrier mRNA at the 2′OH group and the phosphate group position [28] (see Figure 4). The “unevenness” in a polyamine chain arm from TEP may inherently result in a distortion in spatial arrangement and this may seriously impede the overall packaging efficacy. Hence, due to this induced disordering during the packaging of the mRNA, this may have likely resulted in having a smaller size nanomicelle, as observed from both TEM and DLS. While DET and TET may also experience similar reactions at the secondary amine position, from a structural point of view, this effect would not be as severe as compared to TEP, as DET may also present an extension of equal length chains similar to TET when the reaction occurred on the *gamma* positioned secondary amine. Such distortion may also be one of the major reasons why we subsequently observed poorer mRNA delivery from TET-conjugated polyplex nanomicelles in our following observation. In fact, it has also been reported recently that polyamines of different chain lengths would interact differently with nucleic acids, resulting in changes in the structural conformation of the polyamine [29]. Hence, it may be necessary to examine the molecular dynamics of these interactions in the future.

### 3.4. The Proof-of-Concept (POC) Study to Demonstrate the Polyplex Nanomicelles Mediated mRNA Expression in Mouse Brains

**Figure 5:**
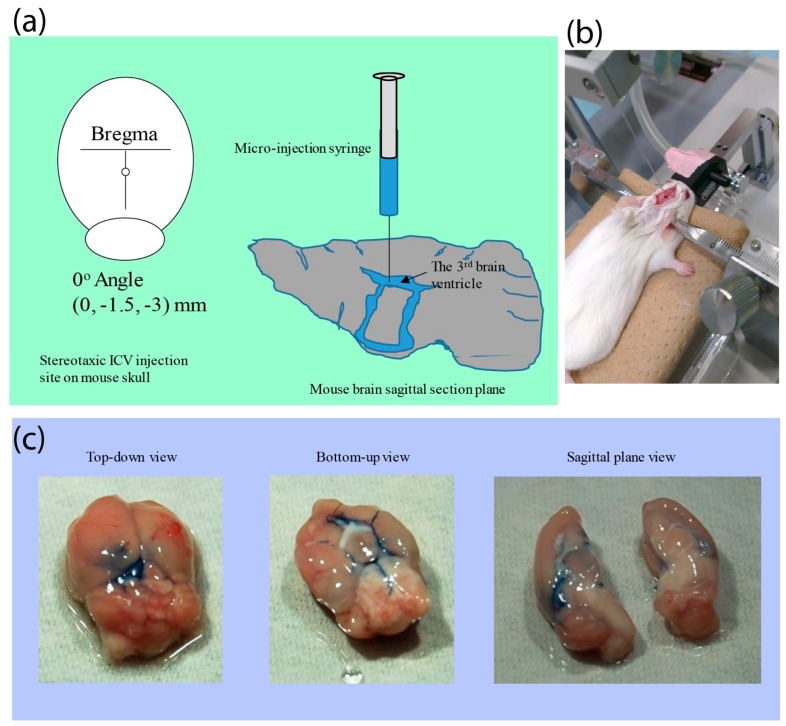
The ICV infusion of trypan blue to demonstrate the mixture solution would flow to whole brain via the connected brain ventricles. (**a**) Illustration of infusion site. (**b**) Intracerebroventricular infusion through micro-syringe. (**c**) Trypan blue flow to whole brain via the connected ventricles.

From the physical characterization, we had confirmed that regardless of the reaction outcome, we were able to condense the polyplex into a nanomicelle of which the ranges were fairly small. In fact, on the basis of this, we decided to deliver *luc2* mRNA as a proof of concept for the future development of mRNA medicine via intracerebroventricular (ICV) infusion. Neurodegenerative diseases and neuron-related disorders are currently affecting millions of people, resulting in tremendous economic and social burdens. However, most of the nanoparticulate-based therapeutics have often suffered from significant drawbacks from the inefficient transport through the brain–blood barrier (BBB). Hence, as a proof of concept, we decided to perform an ICV infusion to circumvent the retardation arising from BBB to evaluate the *luc2* mRNA expression efficiency delivered by polyplex nanomicelles. Prior to the direct administration of *luc2* mRNA polyplex nanomicelles, we had pre-injected trypan blue to evaluate the flow of the mixture solution through whole brain by a third ventricular injection. As shown in Figure 5a, a tiny parietal hole was created on the sagittal suture of skull posterior to the bregma, and subsequently 6 μL of trypan blue was micro-injected to the third ventricle at a perpendicular depth of 3.0 mm (Figure 5b). The mouse brain was removed immediately after the ICV injection and dissected to observe the trypan blue flow in the sagittal plane; imaging showed that the trypan blue was successfully detected throughout the entire brain section through the connected ventricles (Figure 5c). The mRNA polyplex nanomicelles would then be administrated following similar procedure.

**Figure 6:**
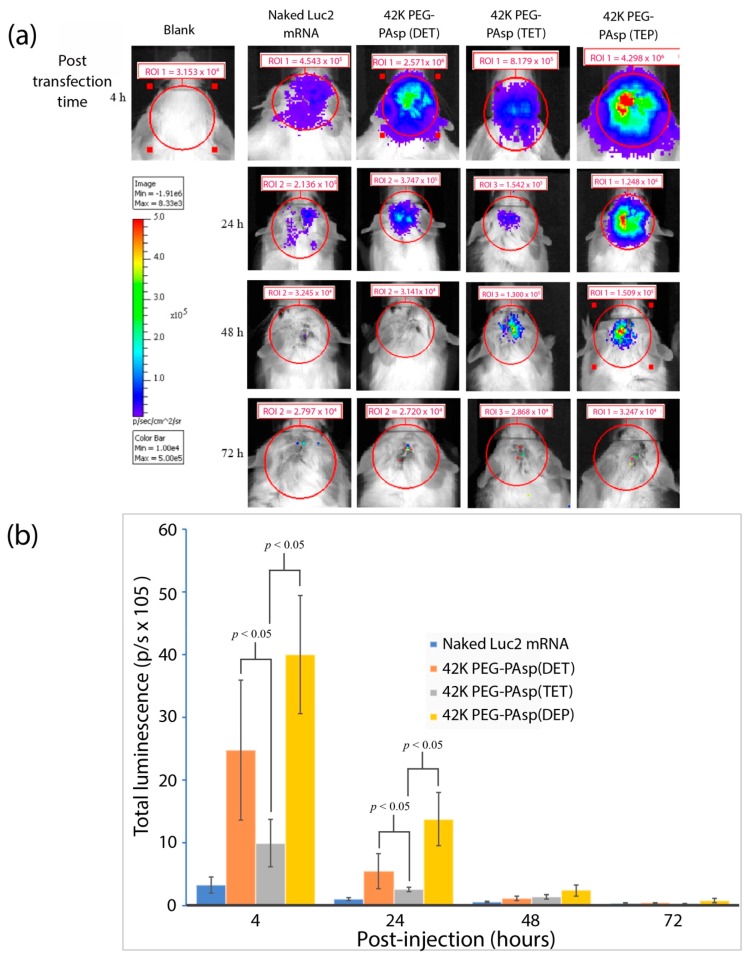
(**a**) IVIS analysis after *Luc2* mRNA polyplex nanomicelles ICV-infusion over a course of 72 h to evaluate the efficacy of signal retention in the brain. (**b**) Total luminescence analysis at 4–72 h post-infusion.

To gain further insights into the *Luc2* mRNA delivery efficiency via an ICV infusion mediated by PEG-polyamine block copolymers, the different repeating units of aminoethylene were administered as earlier mentioned. The *Luc2* mRNA were mixed with 42K PEG-PAsp(DET), 42K PEG-PAsp(TET), and 42K PEG-PAsp(TEP) to assemble into polyplex nanomicelles, after which they were ICV infused in the mouse brains with non-packaged naked *Luc2* mRNA serving as a negative control. The luciferase expression was then monitored by IVIS from 4 to 72 h post-infusion. As shown in Figure 6a, the brain infused with naked *Luc*2 mRNA without PEGylated nanomicelle was observed to have emitted relatively weak luminescence at 4 h post-infusion mark, while the infusion of non-packaged mRNA did not emit any luminescence. Interestingly, luciferase expression from all self-assembly polyplex nanomicelles delivered *Luc*2 mRNA exhibited luminescence signatures throughout the entire 4–48-h post-infusion time-course. Of these, the 42K PEG-PAsp(TEP) showed the most significant luciferase expression when compared with the other three groups. The luminescent photons quantified from IVIS measurements had also demonstrated that luciferase expression in the groups of 42K PEG-PAsp(DET) or 42K PEG-PAsp(TEP) was seemingly superior compared to that in the 42K PEG-PAsp(TET) group throughout the 4–24-h post-infusion time-course (Figure 6b). Once again, this may be in part due to the “uneven arm” hypothesis, as mentioned earlier, and this observation was in agreement with earlier postulation as well as structural analysis. This quantitative data also revealed that the 42K of PEG-PAsp(TEP) possessed the best *Luc2* mRNA delivery efficiency in mouse ICV infusion. Furthermore, in order to correlate the influence of PEG length on the protection of polyplex nanomicelles encapsulated mRNA, we also compared the 12K PEG-PAsp(TEP) and 42K PEG-PAsp(TEP) in the ICV-infusion of *Luc2* mRNA, respectively. Our results had shown the PEG-PAsp(TEP) polyplex nanomicelles with larger PEG molecular weight possessed superior mRNA protection efficiency (Figure S5).

To evaluate the suitability of 42K PEG-PAsp(TEP) as a potential therapeutical mRNA medicine, the cytotoxicity from the administration of 42K PEG-PAsp(TEP) prepared mRNA polyplex nanomicelles was subsequently examined as it was well-established that mRNA possesses strong immunogenicity especially during in vivo administration. To address the subsequent immune responses as elicited from ICV administration, the mouse brains were first infused with naked *Luc2* mRNA or 42K PEG-PAsp(TEP) prepared *Luc2* mRNA polyplex nanomicelles, followed by qRT-PCR to examine the elicited proinflammatory cytokine expression (Figure S6). Our analysis revealed that the 42K PEG-PAsp(TEP) polyplex nanomicelles had indeed presented a significantly lower IL-6 and IFN-β1 expression compared with naked *Luc2* mRNA administration. In conjugation to *Luc2*, we also utilized the 42K PEG-PAsp(TEP) to encapsulate *GFP* mRNA for mRNA ICV-delivery, and our data also suggested that the GFP protein was abundantly expressed in the mouse brain tissue (Figure S7). Once again, this confirmed that the self-assembly of mRNA with 42K PEG-PAsp(TEP) was found to be the most efficient mRNA nanocarrier among the three types of PEG-polyamine block copolymeric systems examined in this work.

To validate the nanocomplex’s low cytotoxicity profile, we had also decided to perform an MTT cytotoxicity assay on the HT-22 mouse’s hippocampal neuronal cell line after 24 h of post-transfection. As shown in Figure S8, we did notice any significant change to the viability of the cells when compared to our control, and this strongly suggested that the nanocomplex has a relatively low cytotoxicity which in turn corroborated well with existing reports [10,20].

## 4. Conclusions

The description here of a PEG-tagged polyamine carrying a mRNA load is a novel concept for the treatment neuron-related diseases, and this paper presents a proof of concept along this route of thought. Our data successfully demonstrated that the PEG-polyamine block copolymers bearing 4 repeating units of aminoethylene possess the most efficient mRNA transgene delivery efficiency among all of our selected block polymers, and also showed very low-leveled pro-inflammatory cytokine stimulatory secretion. Based on the immunohistochemical staining data from the *GFP* mRNA polyplex nanomicelles ICV infusion, 42K PEG-PAsp(TEP) had especially exhibited highly promising efficacy for the transgene delivery into brain tissues; it can potentially serve as a new protection carrier for mRNA medicine for the treatment of neuron disorders. Furthermore, we also compared various lengths of polyamines and hypothesized that the effects from hydrogen bonding as well as the differences in spatial arrangement from the nucleophilic reaction may have contributed to our observed disparity in terms of mRNA delivery as well as retention in vivo. Such correlation in terms of the chemistry with polyamine–RNA interaction had never been reported before in literature to the best of the author’s knowledge. For future work, it would be necessary to further elucidate the precise mechanism of the endosomal escape capability, as well as to perform molecular simulations to help substantiate our hypothesized influence of the ‘uneven’ arm hypothesis. It may be highly plausible that hydrogen bonding may indeed play a larger role than previously expected, and this can provide greater impetus towards appreciating the structural interaction between polyamine and RNA, so as to better design nano-delivery systems based on the above strategy. ROI 1 = 3.154 × 10^4^.

## Supplementary Materials

The following are available online at http://www.mdpi.com/2079-4991/9/1/67/s1, Figure S1: mRNA preparation, Figure S2: NMR spectrum of PEG-PAsp(DET), Figure S3: NMR spectrum of PEG-PAsp(TET), Figure S4: NMR spectrum of PEG-PAsp(TEP), Figure S5: Comparison of PEG length, Figure S6: Proinflammatory cytokine expression, Figure S7: GFP mRNA ICV infusion, Figure S8: MTT assay of HT-22 cells.
