# Supplementary material for: Preparation of Messenger RNA Nanomicelles via Non-Cytotoxic PEG-Polyamine Nanocomplex for Intracerebroventicular Delivery: A Proof-of-Concept Study in Mouse Models
Source: Nanomaterials (Basel). 2019 Jan 5;9(1):67. doi: 10.3390/nano9010067 (PMC6359661; doi:10.3390/nano9010067)
Supplement: Supplementary file 1 [file nanomaterials-09-00067-s001.pdf]

Supporting Information

Assembly of Non-cytotoxic PEG-Polyamine Nanomicelle for mRNA delivery via direct Intracerebroventricular Injection: a proof-of-concept study in Mouse Model

L. Y. Chan[1], Y. L. Khung[2*] and C. Y Lin[1,3*]

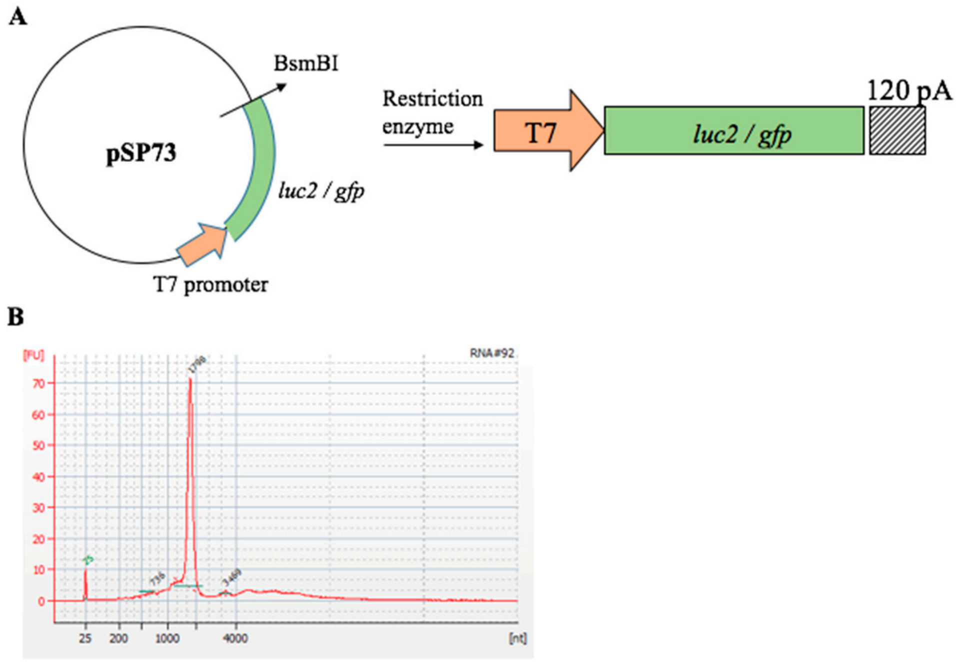

Figure S1. (A) ***Luc2*** **or** ***GFP*** **mRNA were transcribed by linear plasmid DNA containing 120pA sequence digested by BsmBI. (B)** ***Luc2*** **mRNA was analyzed for size and purity with the Agilent RNA 6000 Nano Assay on a BioAnalyzer 2100.**

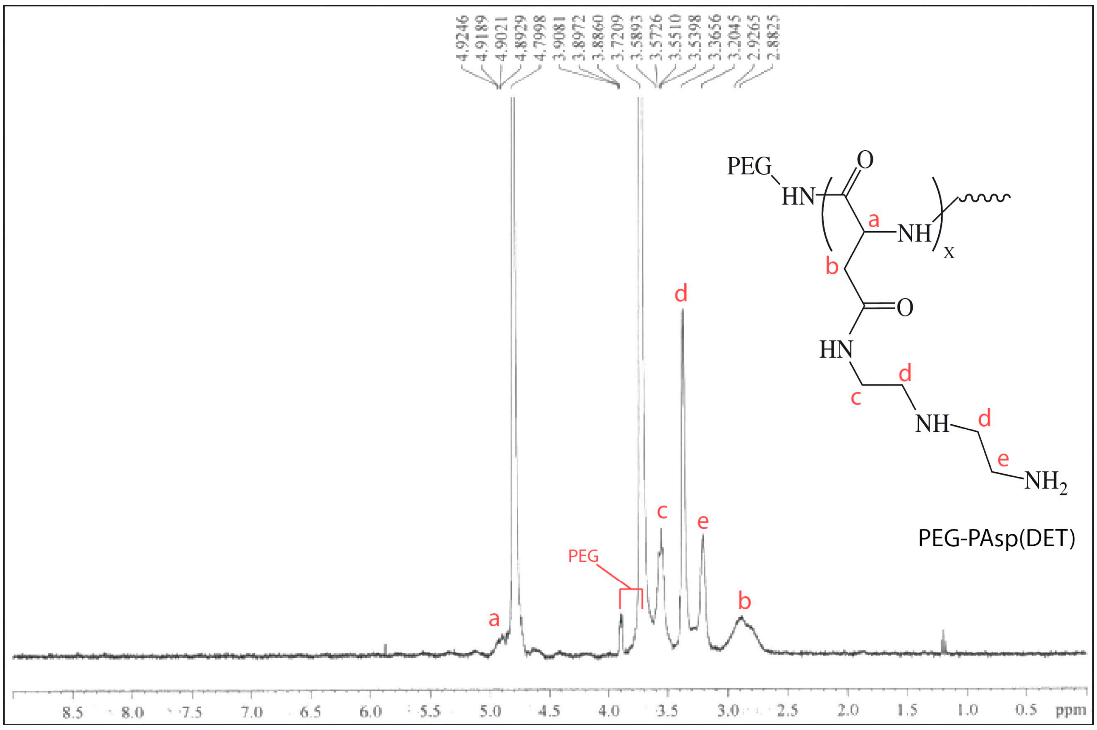

Figure S2. **^{1}H-NMR spectrum for PEG-PAsp(DET)**

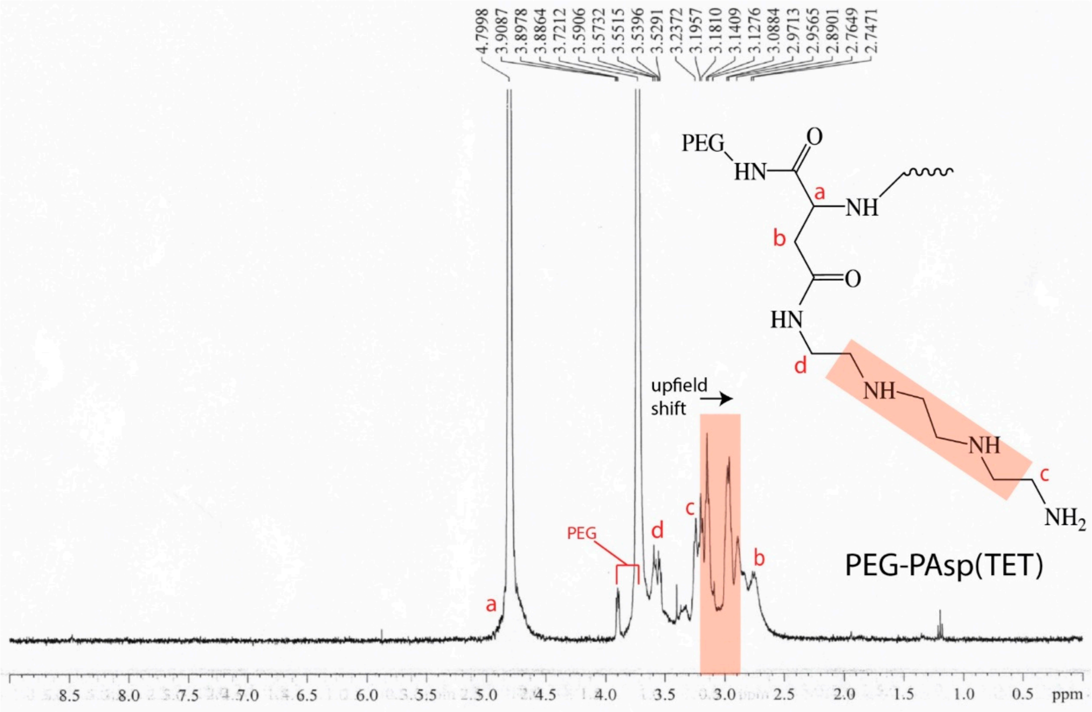

Figure S3. **^{1}H-NMR spectrum for PEG-PAsp(TET)**

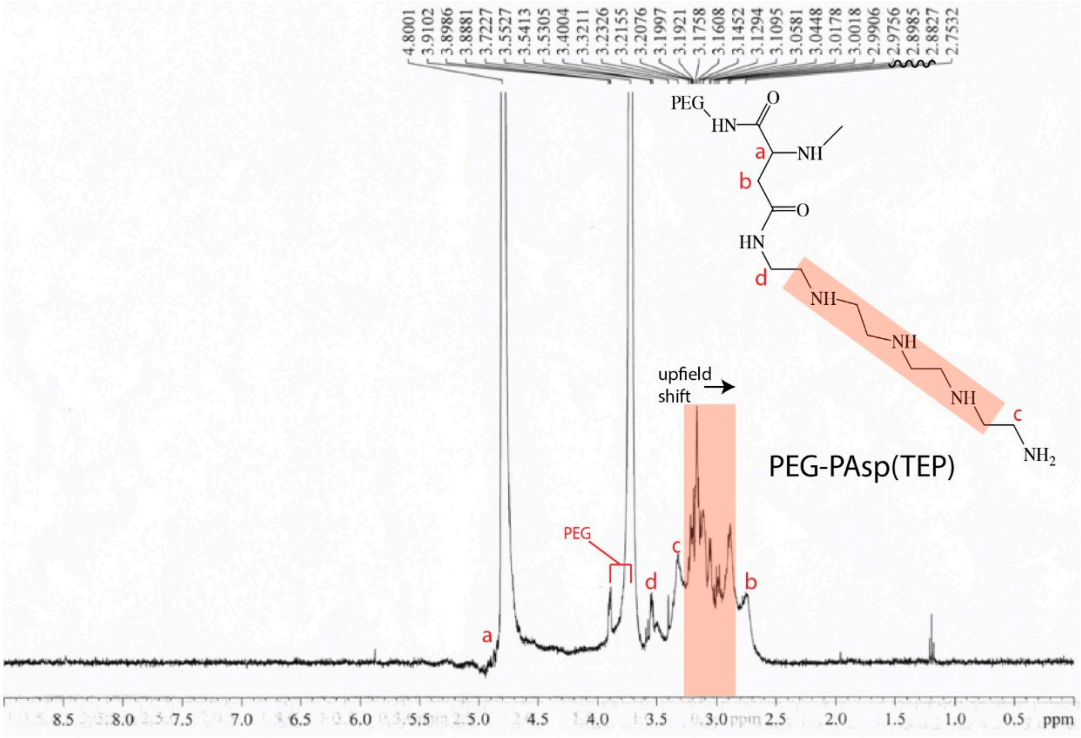

Figure S4. ^{1}**H-NMR spectrum for PEG-PAsp(TEP)**

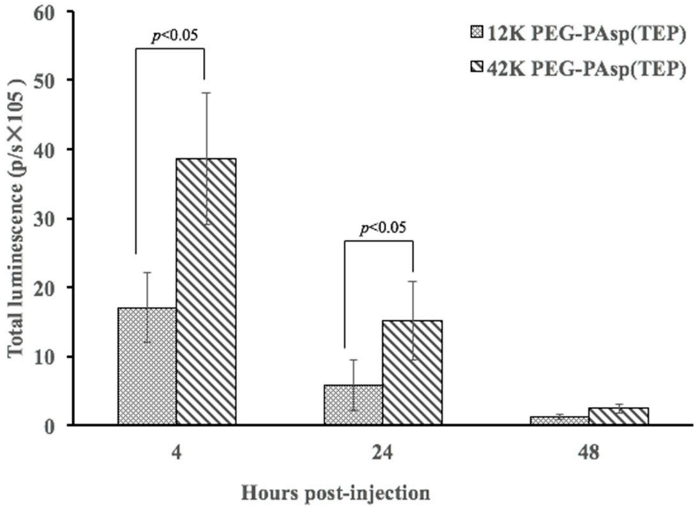

Figure. S5. ***Luc2*** **mRNA was encapsulated by 12K PEG-PAsp(TEP) and 42K PEG-PAsp(TEP) for ICV-infusion in mouse, respectively. Luminescence was measured by IVIS from 4-48 h post-infusion.**

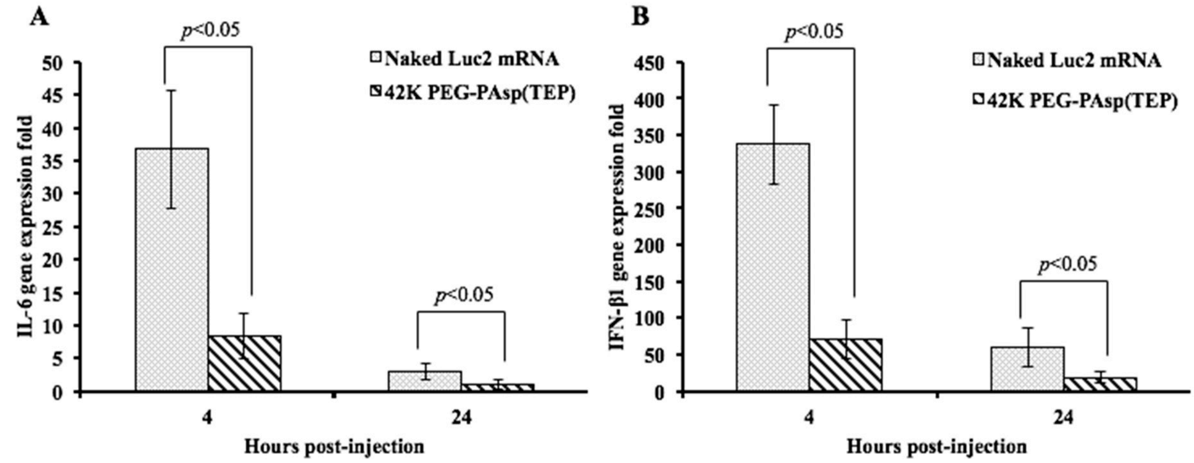

Figure. S6. No significant immune response elicited after ICV injection of self-assembly mRNA nano micelles. (A) IL-6. (B) IFN-b1.

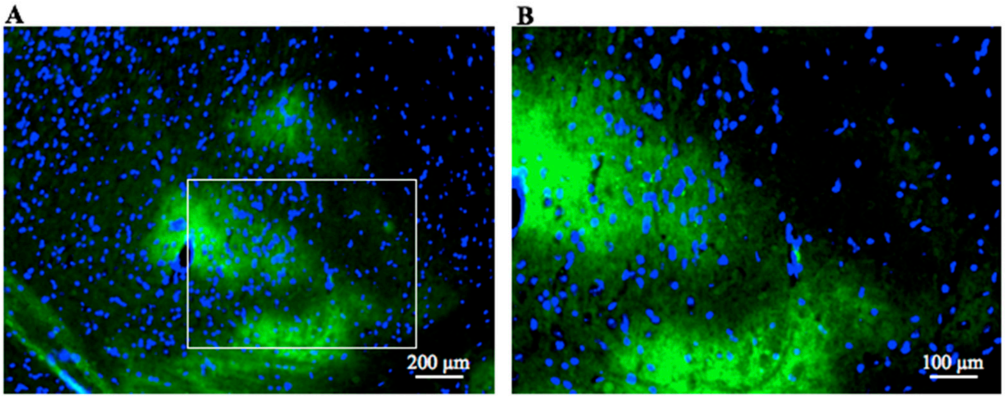

Figure. S7. GFP IHC from ICV injection of self-assembly *GFP* mRNA nano micelles. (A) larger area. (B) Magnified from the white-lined box inset in A.

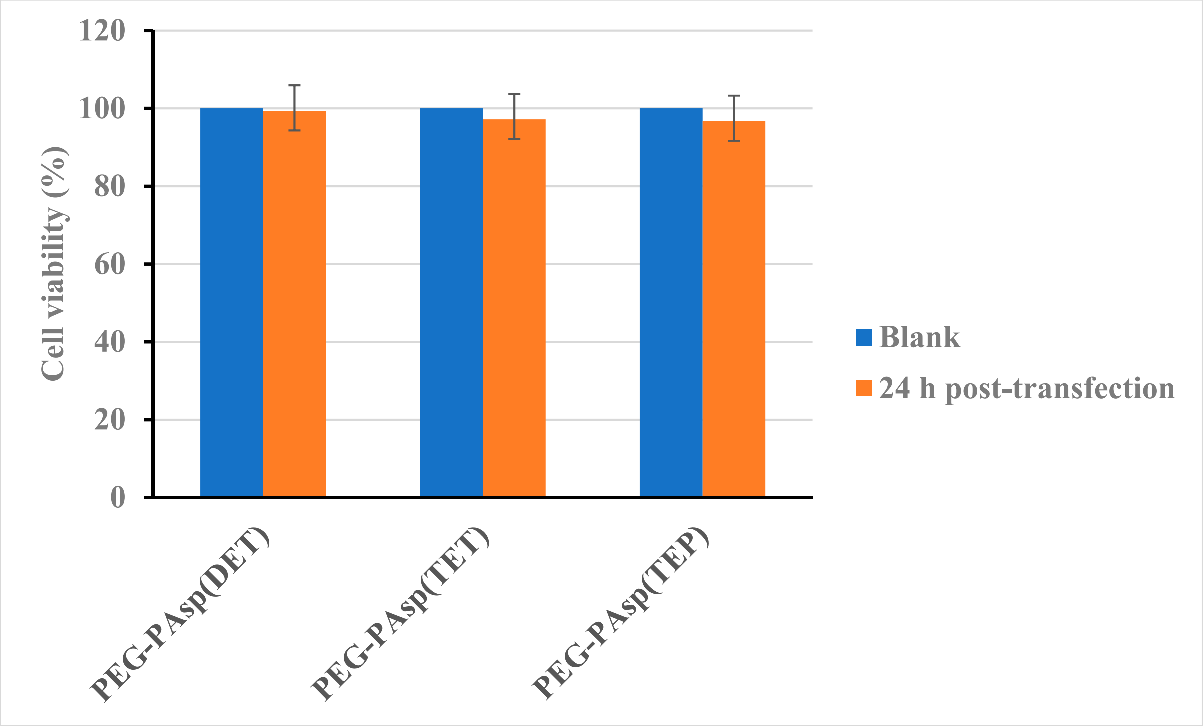

Figure. S8. HT-22 Mouse hippocampal neuronal cell line was transfected with nanomicelles encapsulated with Luc2 mRNA at a N/P ratio of 3, and the measured cytotoxicity with MTT assay after 24 hours post-transfection (hpt). All transfection groups exhibited cell viability > 90%. (N=4)
